# Supplementary material for: p66α Suppresses Breast Cancer Cell Growth and Migration by Acting as Co-Activator of p53
Source: Cells. 2021 Dec 20;10(12):3593. doi: 10.3390/cells10123593 (PMC8700327; doi:10.3390/cells10123593)
Supplement: Supplementary file 1 [file cells-10-03593-s001.zip › cells-1438565-supplementary.pdf]

Supplementary Materials:

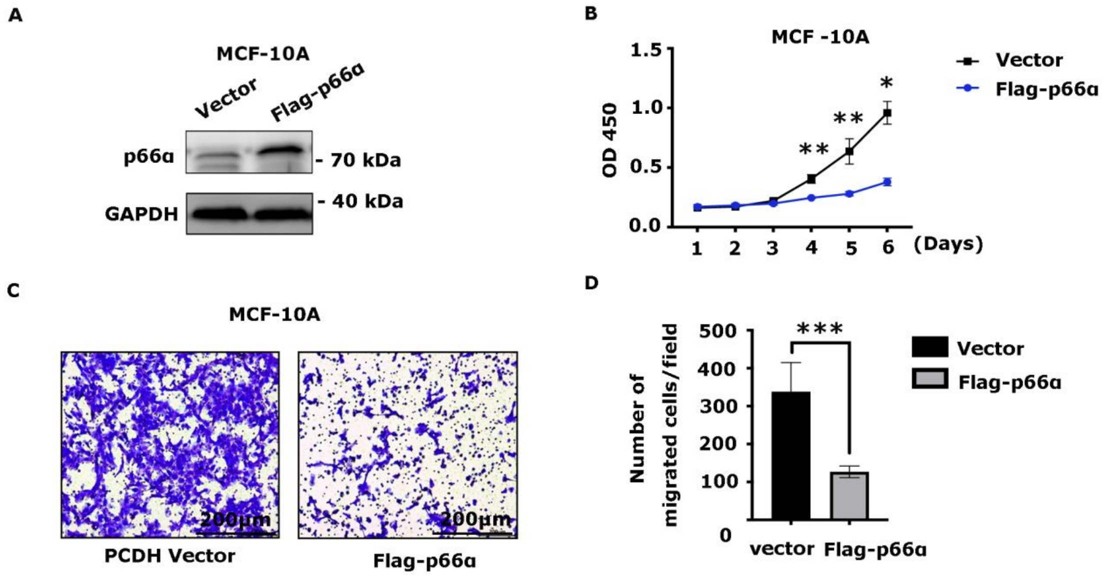

Figure S1. p66α suppresses cell growth and migration in the MCF-10A cell line. (A) Western blot asssays of MCF-10A cell line overexpressing p66α. (B) Cell growth curve of MCF-10A cell line overexpressing p66α. An increase in p66α level impaired cell growth. *P < 0.05, **P <0.01. All data were shown as mean ± S.D. from three independent experiments. (C) Transwell assay shows that an increase of p66α levels reduced migration in MCF-10A cells (10X, Scale bar: 200 μm). (D) Quantification results of cell migration abilities of MCF-10A cells overexpressing p66α. Nine fields were chosen at random for counting statistics. ***P < 0.001.

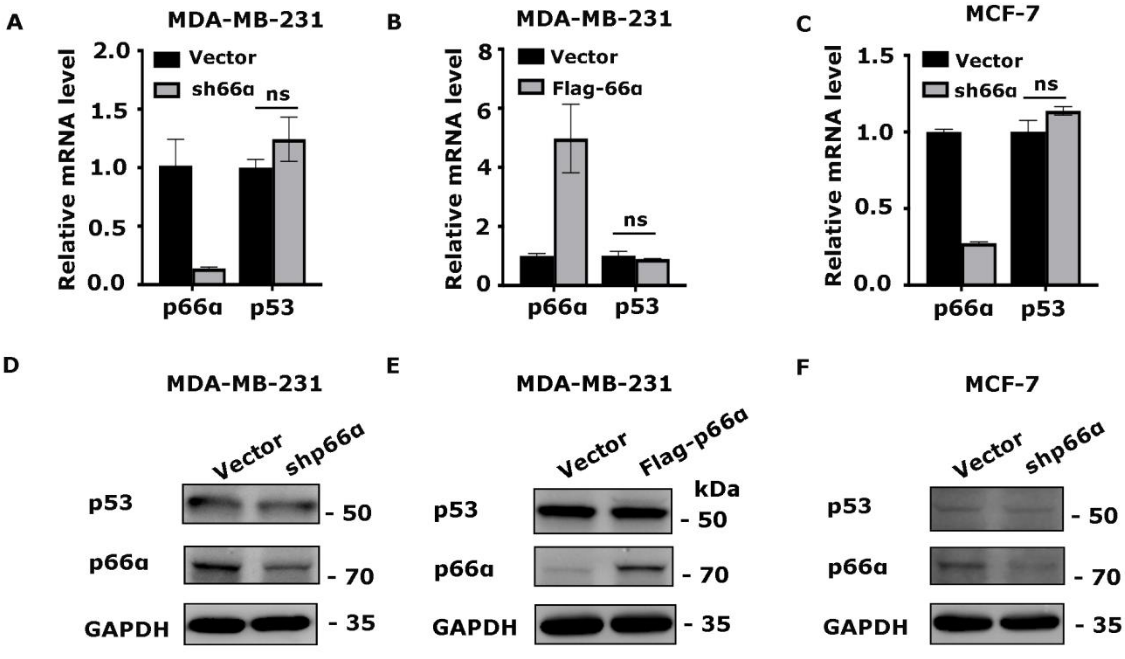

Figure S2. The protein and mRNA level of p53 is not affected by p66α expression or knockdown. (A, B, C) qPCR analysis of the mRNA levels of p53 and p66α in p66α knockdown or overexpressed MDA-MB-231 cells and p66α knockdown MCF-7 cells. (D, E) The western blot analysis of p53 and p66α proteins levels in whole cell extracts, after p66α knockdown or overexpressed MDA-MB-231 cells and p66α knockdown MCF-7 cells.

Table S1: A list of primer sequences applied in qRT-PCR.

Primer	Forward (F)	Reverse (R)
ACTIN	TCACCAACTGGGACGACAT	CACAGCCTGGATAGCAACG
GATAD2A	TGACGTGATTGTGCTCTCCGACAA	TGAAGAGGTCTGGAGTGATGGCTT
TP53	ATGGAGGAGCCGCAGTCAGA	GGCATTCTGGGAGCTTCATC
BAX	TTTTGCTTCAGGGTTTCATC	GACACTCGCTCAGCTTCTTG
GADD45A	AGAGCAGAAGACCGAAAGGA	TGGATCAGGGTGAAGTGGA
NOXA	CAGAGCTGGAAGTCGAGTGT	AGGAGTCCCCTCATGCAAGT
PAI-1	AATCAGCCCACCATGTTCTC	CACCGTCCAGTGCAAAATC

Sequences are listed from 5′ to 3′.

Table S2. A list of primer sequences applied in ChIP qRT-PCR.

Primer	Forward (F)	Reverse (R)
BAX	TAATCCCAGCGCTTTGGAAG	TGCAGAGACCTGGATCTAGCAA
GADD45A	AGCGGAAGAGATCCCTGTGA	CGGGAGGCAGGCAGATG
NOXA	CAGCGTTTGCAGATGGTCAA	CCCCGAAATTACTTCCTTACAAAA
PAI-1	ATTCTCAGGCCAAGGCTATTGG	CCTCGACACCTCCCTCTCT

Sequences are listed from 5′ to 3′.
